# Lignin-Based Copper Nanoparticles for Green and Flexible Electronics

**DOI:** 10.1021/acsaelm.5c02668

**Published:** 2026-03-05

**Authors:** María Salvador, Antonio Santana-Otero, Yilian Fernández-Afonso, Sabino Veintemillas-Verdaguer, André Van Zomeren, Salvador Bertran-Llorens, Alejandro Gutiérrez, María del Puerto Morales

**Affiliations:** † 69570 Instituto de Ciencia de Materiales de Madrid, ICMM/CSIC, C/Sor Juana Inés de la Cruz 3, 28049 Madrid, Spain; ‡ Department of Physics, University of Oviedo, Campus de Viesques, 33204 Gijón, Spain; § The Netherlands Organisation for Applied Scientific Research (TNO), Energy and Materials Transition, Biobased and Circular Technologies Group, P.O. Box 15, 1755 ZG Petten, The Netherlands; ∥ Departamento de Física Aplicada and Instituto Nicolás Cabrera, 16722 Universidad Autónoma de Madrid, C/Francisco Tomás y Valiente, 7, E-28049 Madrid, Spain

**Keywords:** copper nanoparticles, lignin, microwave-assisted polyol synthesis, green synthesis, conductive nanoinks, oxidation resistance, printed electronics

## Abstract

Copper nanoparticles offer a cost-effective and sustainable alternative to silver-based materials for conductive inks in printed and flexible electronics. However, their practical application is often hindered by the need for inert atmospheres and environmentally hazardous reducing agents, but most critically by their rapid postsynthesis oxidation. In this work, we report a green, scalable synthesis of oxidation-resistant metallic Cu nanoparticles via a microwave-assisted polyol method using sodium hypophosphite as a nontoxic reducing agent and lignin as a capping and stabilizing agent. Nanoparticle formation occurs during a final microwave irradiation step as short as 5 min, following a brief homogenization stage, resulting in moderately polydisperse particles with average diameters around 150 nm and without the need for an inert atmosphere. The lignin coating significantly enhances the stability of the particles, maintaining their metallic state for over 120 days in ethanol and 60 days in air. Structural and compositional analyses confirm the effectiveness of lignin in preventing surface oxidation, while electrical conductivity tests show promising values (up to 3.83 × 10^6^ S/m, corresponding to a resistivity of 26.1 μΩ·cm), outperforming commercial references. These results demonstrate the potential of lignin-stabilized copper nanoparticles as eco-friendly conductive fillers for next-generation green and flexible electronics.

## Graphical abstract

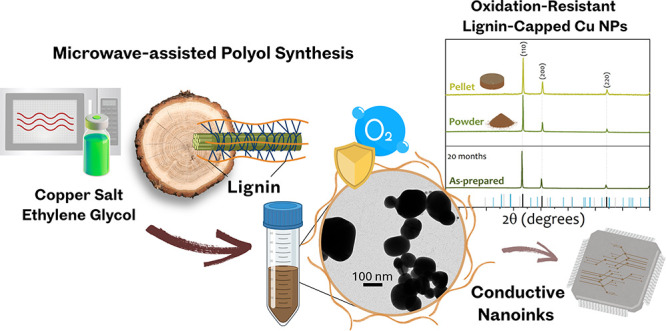

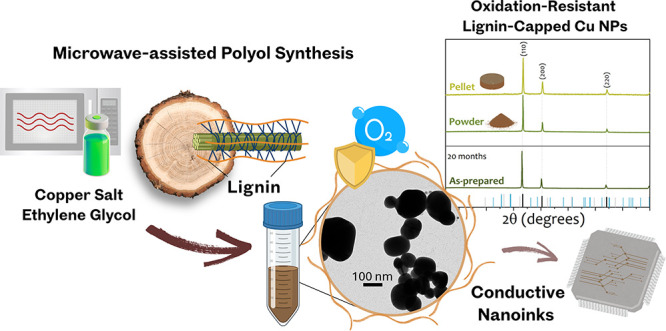

## 1 Introduction

The rapid expansion of the electronics industry, driven by the increasing demand for smart and connected devices, has led to a rising need for advanced materials that are highly efficient. This has placed increasing emphasis on developing greener technologies that combine eco-friendly materials with sustainable, low-cost manufacturing.

Traditional electronics manufacturing often depends on toxic chemicals and energy-intensive methods, contributing significantly to environmental degradation and resource depletion. As a result, current research is increasingly focused on developing alternative materials and methods with a sustainable product life cycle that minimize environmental harm while maintaining or even enhancing performance.^1^ One promising example is printed electronics (PE), which offers an innovative approach that combines traditional printing techniques with advanced materials, and has gained attention for its high production speed, scalability, and environmental benefits.^2,3^ This method reduces material and energy consumption while being able to utilize renewable or biodegradable substrates such as wood, paper, and silk.^4,5^ These substrates impart additional advantages to electronic devices, including lightweight construction and mechanical flexibility,^6^ which are highly sought after in next-generation smart electronics that also promote a greener alternative that facilitates product recycling and helps minimize electronic waste.^7^ Flexible electronics, in particular, benefit greatly from these features, enabling the design of conformable, stretchable, and even foldable devices that can seamlessly integrate into wearable systems, medical sensors, and soft robotics. The compatibility of biodegradable substrates with low-temperature processes also aligns well with the sustainability goals of this emerging field.^8^

Printable conductive materials, or conductive inks, are one of the three key elements of printed electronics technology, along with printing methods and post-treatment processes.^9^ These inks typically consist of a functional core material, a solvent to dissolve or disperse the formulation, and binders or rheological agents. The functional material is responsible for providing the electrical conductivity required for the ink’s intended application. Nanoinks, a specific type of ink, contain metal nanoparticles dispersed in the solution. Metal nanoparticles exhibit unique properties due to their high surface area relative to volume, resulting from their reduced dimensions. Notably, the melting point of these nanomaterials decreases with size, leading to lower sintering and annealing temperatures, both critical steps in forming low-resistance conductive films. These characteristics make metal nanoparticles ideal candidates as fillers for conductive inks.

Despite the flexibility in material choice,^10–14^ silver nanoparticles remain the most widely used due to their high conductivity and long-term resistance to oxidation.^15^ However, silver has notable drawbacks, including its tendency to migrate into dielectric layers under an electric field,^16^ as well as its high cost and environmental impact associated with mining and refining.^17^ In contrast, copper, which is 1000 times more abundant than silver and costs about 1% as much, offers similar conductivity.^18^ Consequently, copper inks are attracting growing interest, with numerous studies demonstrating their ability to form conductive patterns and their potential to significantly reduce the overall environmental impact of PE manufacturing^19,20^ Yet, a major challenge remains their strong tendency to oxidize, particularly at the nanoscale, where high surface-to-volume ratios severely compromise stability and conductivity. Recent analyses emphasize that oxidation mitigation is primarily achieved through surface/interface engineering, ranging from molecular ligands and polymer/biopolymer capping to barrier coatings and core–shell architectures, designed to limit access of reactive species and stabilize the metallic surface.^21^

Overcoming this issue requires the development of synthesis strategies that both prevent oxidation and align with green chemistry principles. Conventional methods, both physical and chemical, often rely on toxic chemicals, require a reducing atmosphere to prevent oxidation, and involve high operational costs due to power consumption, complex equipment, and extended synthesis times.^22–28^ While some techniques can bypass these limitations, they often produce larger particles unsuitable for low-temperature annealing. The polyol method allows to produce metal nanoparticles by reducing metallic salts in various polyalcohols.^29^ Polyols serve multiple roles acting as solvent, reducing agent, and surfactant, limiting particle growth and oxidation while reducing environmental impact.^30,31^ Additionally, their high boiling point promotes higher crystallinity in the samples. Microwave-assisted polyol synthesis offers more uniform heating than conventional methods, leading to more homogeneous nucleation and shorter crystallization times. This approach also improves product yield, reduces reaction time, and enhances reproducibility by preventing temperature gradients during heating. Additionally, microwave technology is further suitable for large-scale production.^32^ Literature reports have also suggested energy-efficiency improvements on the order of ∼30% compared with conventional heating in some systems; however, the magnitude is strongly setup- and scale-dependent, and thus should not be interpreted as a universal value^33^ While microwave assisted synthesis of copper nanoparticles (Cu NPs) has been reported, these methods often rely on toxic reducing or stabilizing agents, such as hydrazine (N_2_H_4_) and sodium borohydride (NaBH_4_).^34,35^ To meet the goals of greener technologies, it is crucial to develop more sustainable synthesis methods that use nontoxic chemicals, eco-friendly solvents, and renewable materials. These advancements would enable Cu NPs to be more widely used in conductive inks, offering both performance and environmental benefits.

In this work, we propose the production of oxidation-resistant, metallic CuNPs for use as conductive fillers in printed electronics via a microwave-assisted polyol method that uses sodium hypophosphite (NaH_2_PO_2_) as reducing agent combined with lignin as a capping and stabilizing agent. Sodium hypophosphite is an abundant, cheap, REACH-registered compound^36^ that is considered as a “nonhazardous substance” for both humans and the environment.^37^ While weak reducing agents typically struggle to fully convert copper salts to metallic Cu, microwave irradiation provides rapid and uniform heating, enabling complete reduction and improved crystallinity.^32^

A key innovation of this work is the use of lignin, a natural, renewable biopolymer byproduct of the paper industry, as a sustainable capping agent. Lignin has been widely explored in sustainable chemistry due to its biodegradability, antioxidant properties, and availability from nonfossil resources. As a capping agent, lignin provides a protective layer around CuNPs, which significantly enhances their resistance to oxidation, even in ambient conditions. Compared to conventional polymer stabilizers, lignin offers a more sustainable and environmentally friendly alternative, both in terms of origin and end-of-life recyclability. Metal nanoparticles stabilized by biopolymers are highly promising for various applications, including biomedical and electronic systems, due to their low toxicity and compatibility with biological systems. Bust most importantly, by capping the particles with lignin, the resulting CuNPs exhibit enhanced oxidation resistance without the need for inert atmospheres or additional stabilizing agents. A representative comparison of recent Cu NP stabilization strategies and reported oxidation stability over time is provided in Table S1; this overview is not restricted to electronic-ink demonstrations, as oxidation progression has been assessed across multiple Cu NP application spaces. These advantages, combined with the simplicity, scalability, and green credentials of the synthesis and the use of renewable materials, make the proposed method a promising alternative for producing Cu NPs as conductive fillers in PE. This work demonstrates a scalable, cost-effective, and eco-friendly solution aligned with the goals of next-generation green electronics.

## 2 Materials and Methods

### 2.1 Materials

Copper­(II) chloride dihydrate (CuCl_2_·2H_2_O, 99%), sodium hypophosphite (NaH_2_PO_2_, >98%), ethylene glycol (C_2_H_6_O_2_, 99%), polyvinylpyrrolidone (PVP40, *M*_w_ 40,000) were obtained from the US company Sigma–Aldrich. were obtained from the US company Sigma–Aldrich. All chemical reagents were used without further purification. Beech wood lignin was obtained by a three-step aqueous acetone organosolv pulping under acidic conditions (a modification of the so-called Fabiola process.)^38^ Commercial Cu NPs, with particle sizes below 100 nm were purchased from Merelex Corp. (reference CU-M-02M-NP.100N). These particles were stabilized in ethylene glycol (EG) through a reductive coating process conducted under strong alkaline conditions and elevated temperature, following a polyol-based synthesis approach previously developed for stabilizing other metal nanoparticles.^39^ In this case, the conventional metal precursor was replaced by commercially available Cu NPs.

### 2.2 Synthesis of the Particles

Copper nanoparticles were synthesized using a polyol reduction process assisted by a microwave reactor (Monowave 450, Anton Paar GmbH). In a typical procedure, 8.71 mmol of CuCl_2_·2H_2_O, 18.4 mmol of NaPO_2_H_2_ and a certain amount of the capping agent (none, PVP40 or lignin) were mixed with 20 mL of EG into a 30 mL MW vial. The microwave-assisted reaction involved heating the mixture in two stages: first, the sample was heated to 70 °C over 10 min and maintained at this temperature for another 10 min. Then, the temperature was increased to 160 °C over the next 10 min and held there for a specific duration. After the reaction, the samples were rapidly cooled to 55 °C. In one additional experiment, the preheating stages were omitted, and the mixture was directly heated to 160 °C as fast as possible and held for 20 min to assess the impact of reaction ramp and homogenization time.

Various synthesis parameters were adjusted to evaluate their influence on the particle properties, including the type and ratio of the capping agent, reaction temperature, and reaction duration (see Table S2 in Supporting Information).

The final product was transferred to a 50 mL Falcon tube and mixed with ethanol to rinse the reactor vial and dilute the mixture. The particles were purified by centrifugation at 7500 rpm (equivalent to 6300 g, calculated for a rotor radius of 100 cm) for 40 min, followed by removal of the supernatant, and the pellet was redispersed in ethanol. This washing process was repeated twice more, with the centrifugation time reduced to 30 min. The samples were then stored in ethanol for further characterization.

### 2.3 Sample Characterization

The particle size, shape, and size distribution were determined by transmission electron microscopy (TEM). The TEM samples were prepared by pouring a few drops of the particle solutions on a carbon-coated grid and leaving it to air-dry. The images were acquired with a 100 keV JEOL-JEM 1010 microscope with a digital camera Gatan model Orius 200 SC. The mean particle size and size distribution were evaluated by measuring the dimension of at least 200 particles with the software ImageJ followed by fitting the data to a log-normal distribution. Scanning electron microscopy (SEM) images were obtained using a field-emission eLINE-PLUS system (Raith GmbH), equipped with an integrated EDX analyzer (Quantax EDS, XFlash 6I30, Bruker), which enabled simultaneous morphological and elemental characterization of the samples.

The crystalline structure of the samples was identified by powder X-ray diffraction (PPXRD). Diffractograms were carried out with a diffractometer (Bruker D8 Advance). The wavelength used is the one corresponding to Cu, while K-Alpha1 [Å] = 1.54060, K-Alpha2 [Å] = 1.54443, and K-Beta [Å] = 1.39225. To be measured, a certain amount of sample was dried on the sample holder before measurement.

For Fourier transform infrared (FTIR) studies, the samples were diluted in KBr at 2% and pressed in pellets. FTIR spectra were acquired between 4000 and 400 cm^–1^ by using a Vertex 70 V FTIR (Bruker) to confirm the presence and nature of the coating. Thermogravimetry analysis (TGA) was performed in an ATD/DSC/TG, Q600 TA Instruments thermobalance by heating the samples under a 100 mL/min nitrogen flow from 25° to 900° at 5° min^–1^.

### 2.4 Oxidation Progression Evaluation

The oxidation behavior of the samples over time was assessed using X-ray diffraction (PXRD) and X-ray photoelectron spectroscopy (XPS). PXRD measurements were performed at multiple time intervals after synthesis to monitor structural changes indicative of oxidation. Samples were stored under different conditions, including suspension in ethanol and direct exposure to ambient air, to evaluate the influence of storage environment on oxidation stability. XPS measurements were carried out using a CLAM4 hemispherical electron energy analyzer and Al Kα radiation (*h*ν = 1486.6 eV) in an ultrahigh-vacuum chamber with a base pressure better than 1 × 10^–9^ mbar. The XPS spectra were fitted by least-squares minimization using Voigt line shapes and a Shirley-type background. As the spectra contain contributions from both metallic Cu and CuO, the fitting procedure was based on a two-component model. The metallic Cu contribution was described by two Voigt components corresponding to the Cu 2p3/2 and Cu 2p1/2 core levels. In contrast, the CuO contribution exhibits a more complex line shape due to the presence of multiplet features and satellite structures. To account for this, the CuO component was constructed using the parameters obtained from a prior fit of a reference CuO sample. In the fitting of mixed Cu/CuO spectra, the CuO line shape was guided by the relative peak positions and internal intensity ratios determined from the reference CuO fit, while allowing for limited variations to achieve an optimal agreement with the experimental data. The final fit was obtained as the sum of the metallic Cu and CuO contributions. In addition, the thickness of the CuO surface shell was estimated from the attenuation of the metallic Cu 2p signal using a standard overlayer/substrate model for a uniform oxide overlayer on metallic Cu. For normal emission, the oxide thickness *t* can be expressed as

$$t=\lambda_{\mathrm{CuO}}\ln\left(1+\frac{I_{\mathrm{CuO}}n_{\mathrm{CuO}}\lambda_{\mathrm{Cu}}}{I_{\mathrm{Cu}}n_{\mathrm{Cu}}\lambda_{\mathrm{CuO}}}\right)$$ 1

where *I*_CuO_ and *I*_Cu_ are the fitted Cu 2p intensities assigned to CuO (including shakeup satellites) and metallic Cu, respectively; *n*_CuO_ and *n*_Cu_ are the Cu atomic densities in CuO and metallic Cu; and λ_CuO_ and λ_Cu_ are the inelastic mean free paths (IMFP) for electrons of kinetic energy *E*_*k*_ ≈ 550 eV traveling through CuO and Cu. IMFP values were taken from the TPP-2 M formalism (λ_CuO_ ≈ 1.22 nm and λ_Cu_ ≈ 1.00 nm), using typical densities for Cu and CuO.

### 2.5 Reproducibility of the Synthesis

To evaluate the reproducibility of the optimized synthesis protocol, the same microwave-assisted polyol reaction was repeated five times under identical experimental conditions. These replicates were carried out using the formulation that exhibited the best performance in terms of particle stability, oxidation resistance, and conductivity. All synthesis parameters, including reagent concentrations, microwave heating profile, and reaction volume, were kept constant to assess the consistency of the process. The resulting Cu NPs were characterized using TEM and PXRD to compare particle morphology, size distribution, and crystalline structure across the five batches.

### 2.6 Conductivity Measurements

To evaluate the electrical properties of the synthesized Cu NPs, conductivity measurements were performed on compressed powder pellets 5 weeks postsynthesis. The as-synthesized samples were first dried to obtain a powder, which was then compressed into pellets using a PerkinElmer hydraulic press. The pellets were prepared following a standard KBr pelletization protocol: the dried powder was pressed into 13 mm diameter and 0.3–0.5 mm thick disks under a pressure of 3 tons, maintained for 5 min to ensure homogeneous and consistent compaction. Electrical conductivity was measured using a four-point probe system (Ossila Ltd., Sheffield, UK). Measurements were conducted at room temperature.

## 3 Results and Discussion

### 3.1 Synthesis and Characterization of the Metallic Copper Nanoparticles

Copper nanoparticles were synthesized via a microwave-assisted polyol method using three different stabilizing conditions: no capping agent (Cu), polyvinylpyrrolidone PVP40 (CuP), and lignin (CuL). The sample names in parentheses will be used hereafter. This synthetic approach offers a notable advantage, as the polyol medium (ethylene glycol) serves not only as the solvent but also helps with the reduction, facilitating the electron transfer process required for the reduction of copper ions in solution.^40^ Moreover, the addition of surfactants or capping agents plays a crucial role in controlling nanoparticle growth within the nanometer range and minimizing polydispersity.

TEM analysis, shown in Figure 1, reveals distinct differences in particle size and distribution among the three samples, with quantitative values summarized in Table 1. The sample without capping agents (Cu) exhibits the largest average particle size. In contrast, the samples synthesized with PVP and lignin display significantly smaller average sizes, being 97 and 114 nm, respectively. However, the particle size distribution is substantially narrower in the case of lignin, with a standard deviation much smaller compared to both CuP and Cu, indicating a more homogeneous particle population. These findings suggest that the capping agents, PVP and lignin, play a crucial role in controlling the combination of nucleation, growth and agglomeration that occur during synthesis by adsorbing onto the nanoparticle surface and interacting between themselves once nucleated. PVP was the most successful in the control of particle size and lignin in the production of less agglomerated samples. This trend is further corroborated by the SEM images (see Figure 2), where the CuL sample shows a visibly narrower size distribution and reduced clustering compared to the uncapped Cu sample. These observations reinforce the conclusion that lignin contributes to the production of more homogeneously dispersed nanoparticles. In the context of printed electronics, although sub-50 nm Cu nanoinks are advantageous for inkjet printing and ultrafine features, larger nanoparticles can be fully compatible with screen/stencil printing and coating routes used for thicker conductive traces. In such applications, oxidation stability and the ability to form effective interparticle contacts after consolidation/sintering are often more critical than primary particle size alone.^41–43^

**Figure 1:**
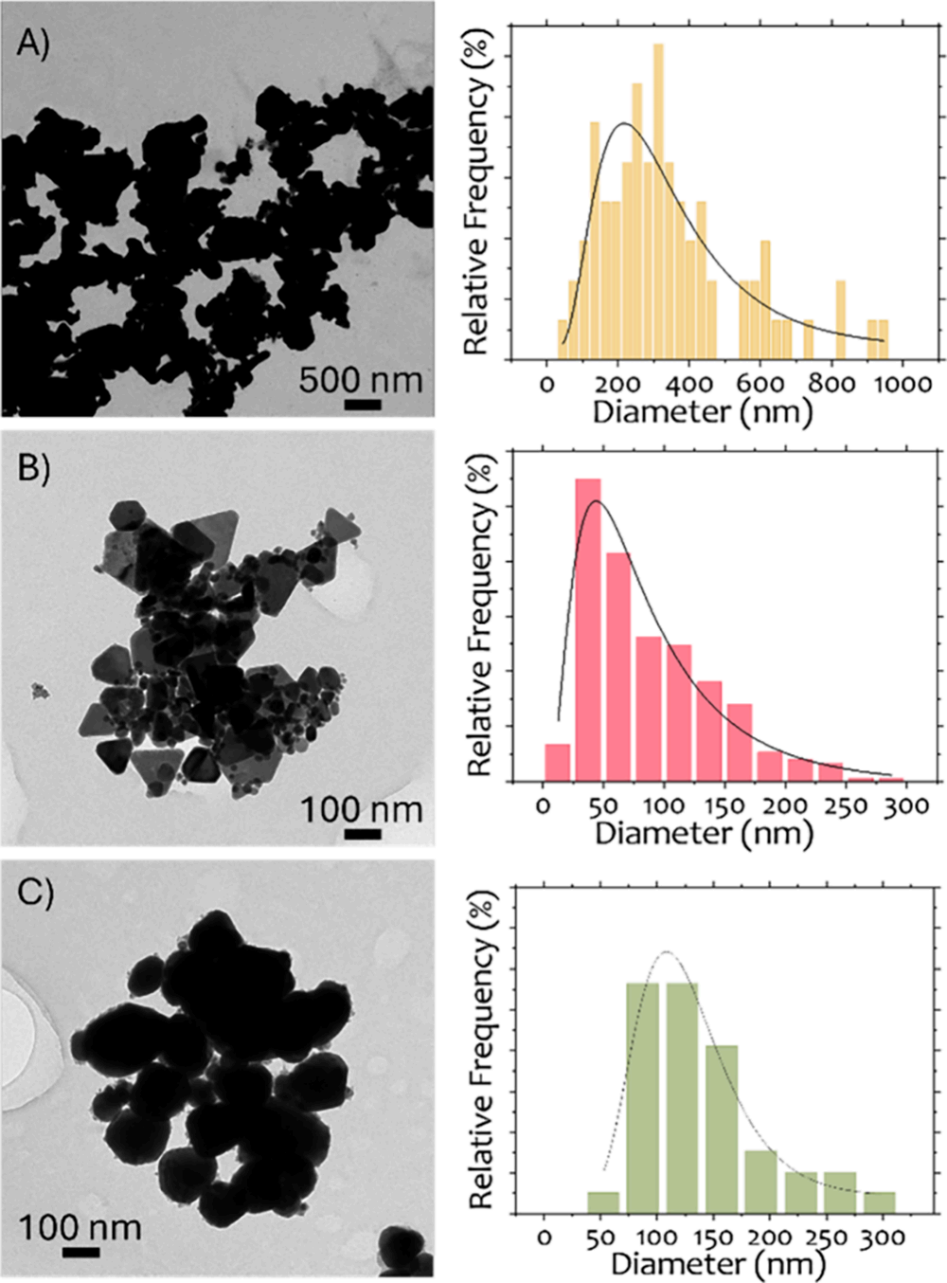
TEM images and particle size distributions of the samples (A) Cu, (B) CuP, and (C) CuL.

**Table 1:** Summary of the Copper Nanoparticle Synthesized under Different Stabilizing Conditions, Indicating the Average Particle Size (*D*_TEM_) and Its Standard Deviation Determined by TEM (σ_TEM_), the Crystallite Size Estimated from PXRD Spectrum (D_PXRD_) and Its Standard Deviation (σ_PXRD_), and the *a* Parameter and Its Standard Deviation (σ_*a*_)

sample	capping agent	*D*_TEM_ (nm)	σ_TEM_ (nm)	*D*_PXRD_ (nm)	σ_PXRD_ (nm)	*a* value (Å)	σ_*a*_ (Å)
Cu	NA	354	220	72	12	3.611	0.0003
CuP	PVP40	97	81	16	6	3.614	0.0003
CuL	Lignin	114	28	64	42	3.614	0.0001

**Figure 2:**
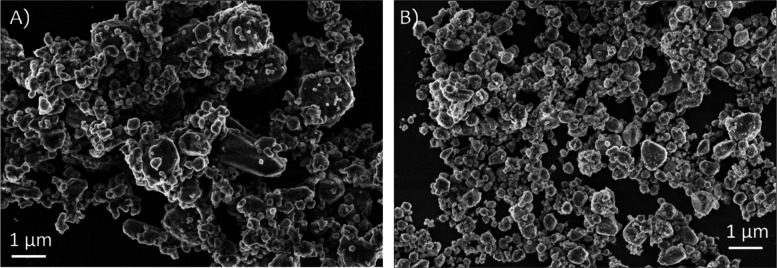
SEM images of copper nanoparticles synthesized (A) without capping agent (Cu) and (B) with lignin as stabilizer (CuL).

PXRD patterns in Figure 3 confirm the influence of the capping agents on the chemical stability of the samples. The CuL sample shows only peaks corresponding to face-centered cubic metallic copper, indicating that the nanoparticles remain unoxidized. In contrast, both Cu and CuP samples show incipient additional diffraction peaks corresponding to either CuO or Cu_2_O, indicating the presence of these oxides. These results demonstrate that lignin acts as an effective antioxidant capping agent, stabilizing the metallic copper phase even under ambient conditions. Rietveld refinement of the PXRD data yield lattice parameter values in good agreement with the standard face-centered cubic structure of metallic copper (see Table 1), confirming the high crystallinity and phase purity of the synthesized nanoparticles. Additionally, crystallite sizes estimated from it were 41 nm for Cu, 16 nm for CuP, and 64 nm for CuL. These values are consistent with the particle size trends observed by TEM, where the presence of the capping agents reduced polydispersity and helped to maintain nanoscale dimensions.

**Figure 3:**
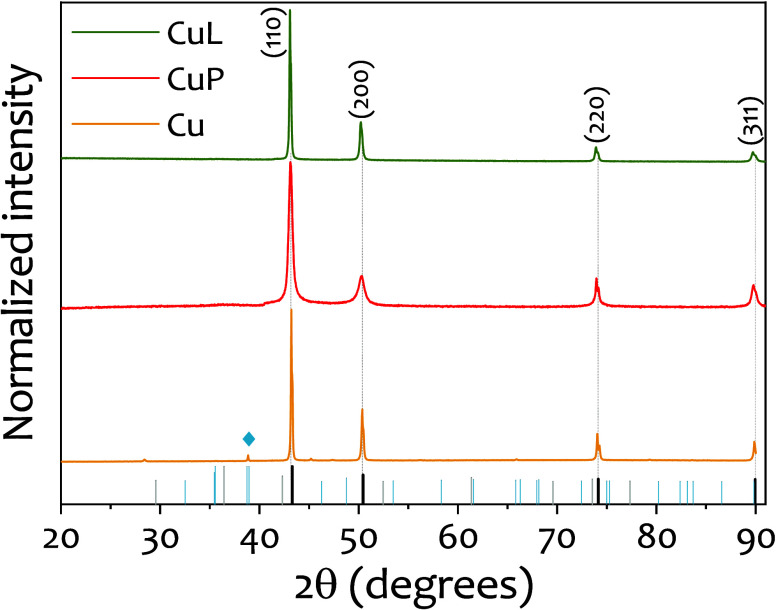
PXRD diffractograms of the samples obtained with no capping agent (Cu), PVP40 (CuP), and lignin (CuL). PXRD patterns for Cu (PDF 00-004-0836), CuO (PDF 00-041-0254), and Cu_2_O (PDF 00-005-0667) are represented in in black, blue, and gray, respectively. Symbols ⧫indicate the indexed CuO diffraction peaks.

Thermal stability and organic content of the Cu NPs were assessed via TGA and DTA, shown in Figure 4A. Both samples, CuP and CuL, exhibit distinct weight loss events associated with decomposition of the capping agents and oxidation of copper.

**Figure 4:**
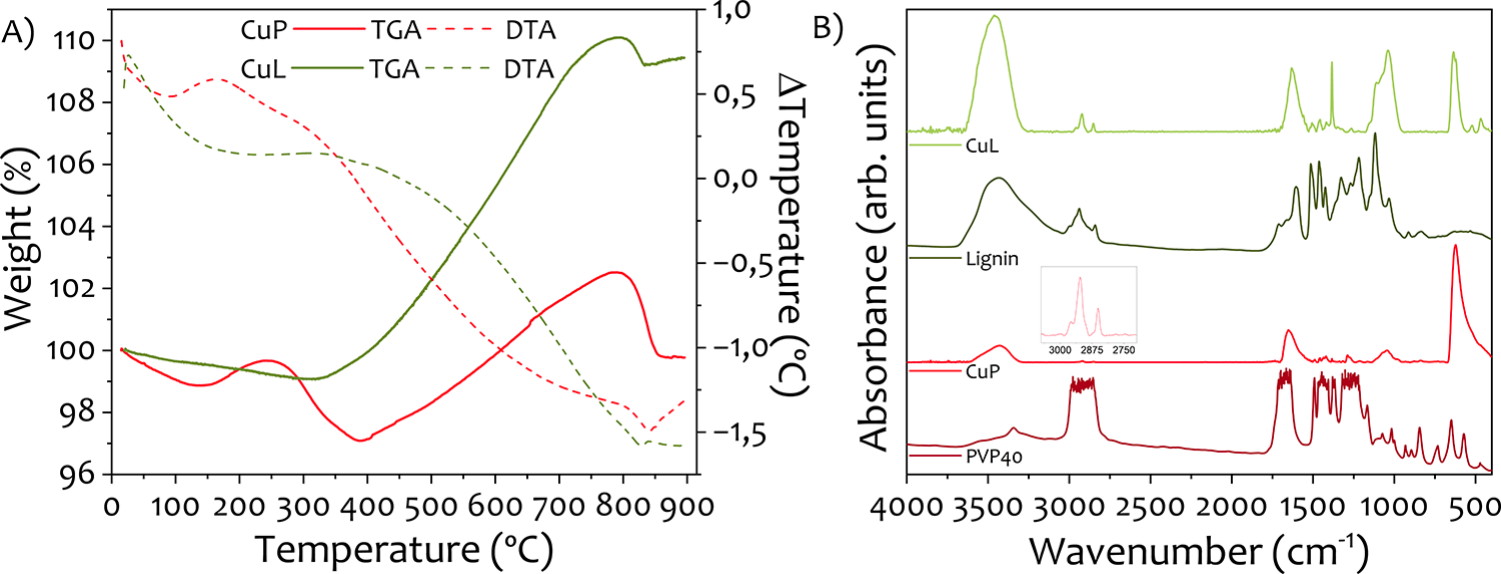
(A) TGA analysis in nitrogen atmosphere and (B) FTIR spectra of the samples CuP and CuL.

The TGA curve of the CuP sample shows an initial minor weight loss near 150 °C, likely due to the evaporation of adsorbed moisture or residual solvents. This is followed by a slight mass gain up to approximately 250 °C, which might be associated with surface processes such as the formation of a transient passivation layer. Studies have shown that Cu NPs can begin to oxidize at temperatures as low as 150–300 °C, potentially leading to a measurable weight increase as a thin copper oxide layer forms on the nanoparticle surfaces.^44^ Following the mass gain, a more pronounced mass loss starts at 250 °C, peaking at around 400 °C. This mass loss can probably be explained by the decomposition of the carbonic backbone and pyrrolidone group. According to literature, PVP begins to decompose above 350 °C, with major degradation occurring between 400 and 500 °C, depending on molecular weight and processing conditions.^45,46^ Beyond this temperature, the residual mass gradually increases, reaching a maximum near 800 °C. This mass gain is likely related to oxidation of the copper core, induced by the catalytic activity of carbonaceous residues derived from the decomposition of the organic coating.

In the CuL sample, the decomposition profile is simpler and reflects the more gradual degradation behavior of lignin. Lignin decomposes slower and over a broader temperature range (200–500 °C),^47^ which is clearly seen in the TGA. A very subtle mass loss occurs near 100 °C, likely corresponding to solvent desorption. This is followed by a slow and steady weight loss, peaking around 350 °C. The degradation of lignin’s polymeric structure begins at relatively low temperatures (200–275 °C), with the main decomposition process occurring around 400 °C. During this stage, a complex mixture of aromatic hydrocarbons, phenolics, hydroxyphenolics, and guaiacyl-/syringyl-type compounds are released, many containing phenolic −OH groups.^48^ In the TGA profile, this decomposition appears to overlap with the oxidation of the metallic copper core, as evidenced by a renewed mass gain starting at around 350 °C and peaking again near 800 °C, similar to that seen for the CuP sample.

In both cases, the thermal degradation of capping agents such as PVP and lignin does not leave behind inert residues. Instead, they release small quantities of CO_2_, CO, and volatile oxygenated species (e.g., aldehydes, ketones), which may act as mild oxidizing agents and promote the oxidation of metallic copper (Cu^0^ → Cu^+^/Cu^2^^+^). Simultaneously, the residual carbonaceous matter derived from incomplete degradation can remain in the nanoparticle surface. Although carbon-based char is often regarded as a protective layer, certain carbon–oxygen complexes or defect-rich carbon residues can promote surface reactions that accelerate copper oxidation under inert or semi-inert conditions The CuL sample shows a larger final mass increase, exceeding 100% of the original mass, compared to CuP. This observation suggests that lignin may generate a greater amount of carbonaceous residue than PVP, in agreement with previous reports on its high char-forming capacity, often exploited in flame-retardant materials.^49^ This higher char content may facilitate the formation of reactive interfaces that enhance copper oxidation under thermal stress.

To investigate the interaction between the stabilizing agents and the nanoparticle surfaces, FTIR spectra were recorded for the pure PVP and lignin samples, as well as for the Cu NPs stabilized with each compound (CuP and CuL, respectively). The spectra are shown in Figure 4B.

The FTIR spectrum of pure PVP displays characteristic absorption bands at 3550–3230 cm^–1^ (broad O–H stretching due to hydrogen bonding), 2950 cm^–1^ (C–H stretching), and around 1655 cm^–1^ (C=O stretching). Additional signals are observed in the regions 1305–1200, 1190–1170, and 1145–1130 cm^–1^, corresponding to C–N stretching. Most of these bands are still present in the CuP spectrum but appear with slight shifts and reduced intensity, as shown in the inset of Figure 4B. This suggests interaction between the PVP chains and the copper nanoparticle surface, likely via coordination of the carbonyl oxygen to the copper surface.

Lignins contain diverse functional groups, such as hydroxyl, methoxyl, carbonyl, and carboxyl, whose quantities vary based on their origin and isolation process. These groups, coupled with the unique network structure of lignins, confer distinct functional properties to the material.^47^ The spectrum of pure lignin shows a broad absorption band at 3410–3460 cm^–1^, attributed to hydroxyl groups in phenolic and aliphatic structures, and bands centered around 2938 and 2842 cm^–1^, which arise from CH stretching in aromatic methoxyl groups and in methyl and methylene groups of the side chains. Additionally, distinct peaks at 1595 and 1510 cm^–1^ are assigned to aromatic C=C stretching, and bands in the 1200–1000 cm^–1^ region correspond to C–O and phenolic group vibrations. These characteristic bands are also observed in the CuL spectrum, with slight shifts and decreased intensity, consistent with adsorption of lignin onto the nanoparticle surface. Importantly, the −OH stretching region (3400 cm^–1^) remains in the CuL spectrum, albeit with reduced intensity, indicating the retention of phenolic groups from lignin.^50^ This supports the successful integration of lignin onto the nanoparticle surface and its role in stabilizing the metallic copper phase. By contrast, phosphate species^51^ originating from sodium hypophosphite would typically show strong P=O and P–O stretching bands in the 1150–1000 cm^–1^ region, which are not detected in the FTIR spectrum of CuL. Furthermore, SEM–EDX analysis of the CuL nanoparticles and XPS measurements in the P 2p region (Figure S1 in Supporting Information) confirmed the absence of phosphorus signals, ruling out phosphate-based surface species.

Additionally, both CuP and CuL spectra show weak bands around 600 cm^–1^, which could be attributed to Cu–O stretching modes. However, no crystalline copper oxide phases were detected by PXRD in these samples. FTIR spectroscopy provides information about short-range order and functional group interactions, while PXRD gives insights into long-range crystallographic structure. Thus, observing bands in FTIR but not in PXRD suggests that if any oxide is present, it is either amorphous or forms an extremely thin surface layer, insufficient to produce detectable diffraction peaks. In summary, these FTIR results confirm the presence of both PVP and lignin on the nanoparticle surfaces, indicating effective surface stabilization.

To further investigate the role of the capping agent, additional syntheses were conducted using varying concentrations of lignin and PVP. The objective was to evaluate how the amount of stabilizer influences the formation and phase purity of the Cu NPs. PXRD measurements were performed on these samples to assess potential differences in crystalline and oxidation states. The results (see Figure 5A) revealed that CuL samples exhibited no variation in their PXRD patterns, all showing pure metallic copper with no detectable copper oxide phases, regardless of lignin concentration (twice (CuL^+^) and half (CuL^–^) of the lignin in CuL.) In contrast, CuP samples demonstrated a dependency on PVP concentration: the sample with twice the amount of PVP (CuP^+^) displayed peaks corresponding to Cu_2_O, indicating partial oxidation, while the sample with half the amount of PVP content (CuP^–^) retained a pure metallic copper phase. These results suggest that excess PVP may hinder the reduction efficiency during nanoparticle formation. PVP functions as a steric stabilizer, adsorbing onto the particle surface and forming a protective polymeric layer. However, when present in large amounts, this layer can become excessively dense, potentially impeding the access of the reducing agent to the copper ions. As a consequence, the reduction process may become incomplete, leading to the presence of residual Cu^+^ or Cu^2^^+^ species. These unreduced species can subsequently oxidize, resulting in the formation of copper oxide phases observed in the PXRD patterns of the CuP^+^ sample. Interestingly, this hindrance effect was not observed for lignin, even at higher concentrations. A possible explanation lies in the substantially lower molecular weight of lignin (∼3 kDa) compared to PVP (∼40 kDa), which results in a less bulky and more loosely packed stabilizing layer on the nanoparticle surface. This more open structure likely facilitates better accessibility of the reducing agent to the copper ions during synthesis, enabling complete reduction and limiting the formation of copper oxides. Moreover, lignin’s irregular, branched structure and multifunctional chemical groups may promote more dynamic interactions at the particle surface, avoiding the formation of diffusion barriers that could otherwise inhibit reduction. These results suggest that molecular weight can significantly influence surface layer density and reactivity. Future studies will aim to systematically assess this parameter by comparing different biobased or synthetic stabilizers with molecular weights closer to that of lignin, in order to better isolate and understand the role of polymer chain length in copper nanoparticle formation and oxidation resistance.

**Figure 5:**
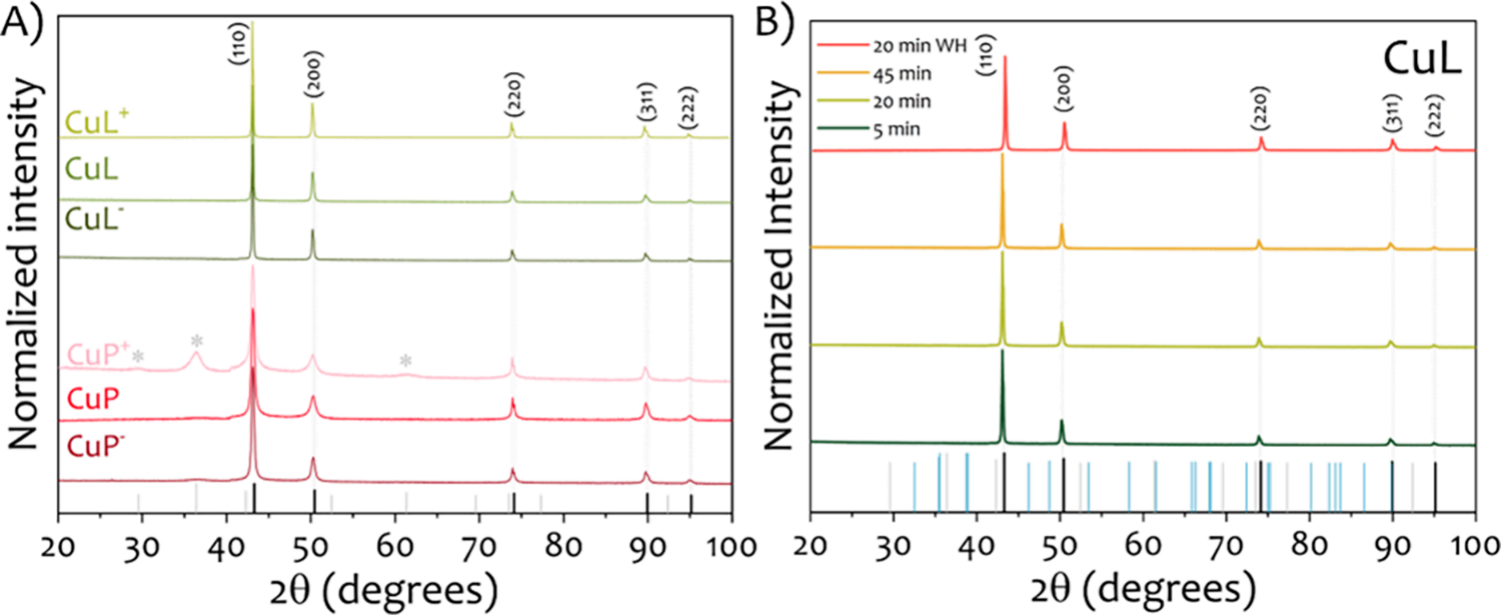
(A) PXRD diffractograms of Cu NPs synthesized with different amounts of both lignin and capping agents. Samples CuL^+^, CuL, and CuL^–^ correspond to high, standard, and low concentrations of lignin, respectively. Similarly, CuP^+^, CuP, and CuP^–^ represent high, standard, and low concentrations of PVP. PXRD diffractograms of the samples obtained with different amounts of capping agents, both PVP and lignin. (B) PXRD diffractograms of the samples obtained at different reaction times (5, 20, and 45 min) when lignin is used as capping agent. The sample labeled “20 min WH” corresponds to a synthesis performed without the preheating and homogenization steps, where microwave heating was applied directly until 70 °C was reached and then maintained for 20 min. PXRD patterns for Cu (PDF 00-004-0836), CuO (PDF 00-041-0254), and Cu_2_O (PDF 00-005-0667) are represented in in black, blue, and gray, respectively. Symbols * indicate the indexed Cu_2_O diffraction peaks.

Finally, to assess the impact of reaction time on phase formation, CuL samples were synthesized using microwave irradiation for 5, 20, and 45 min. PXRD analysis in Figure 5B confirmed that all reaction durations resulted in the formation of metallic copper as the dominant phase, with no detectable signals from any copper oxides. To further validate that the reduction step occurs rapidly, a control synthesis was performed in which the preheating and homogenization stages were omitted. In this case, microwave heating was applied directly until 70 °C was reached and then maintained for 20 min (“20 min WH” in Figure 5B). The PXRD diffractogram of this sample also showed pure metallic copper, confirming that the reduction and crystallization processes occur during the final microwave step and do not depend on earlier heating stages.

This suggests that lignin effectively facilitates the rapid and complete reduction of copper ions, even at shorter synthesis times, and contributes to the stabilization of the metallic copper phase. These results highlight the strength of the method and the key role of lignin in maintaining a reducing environment throughout the process. Moreover, the ability to achieve full reduction within just 5 min presents an opportunity to reduce energy consumption during synthesis, further enhancing the sustainability of the approach.

### 3.2 Oxidation Progression

The progression of oxidation in the synthesized Cu NPs was investigated over time under different storage conditions, aiming to reflect typical shelf life durations of ink precursors, given their well-known susceptibility to oxidation.^52^ Figure 6 presents the evolution of PXRD patterns for both CuP and CuL samples analyzed at various time points, expressed as days (“d”) or months (“m”). The nomenclature used in the figure reflects the storage environment: samples without any additional label were stored in ethanol immediately after synthesis; those labeled “air” were stored as dry powders under ambient conditions; and those marked with “P” were compressed into pellets and stored in air.

**Figure 6:**
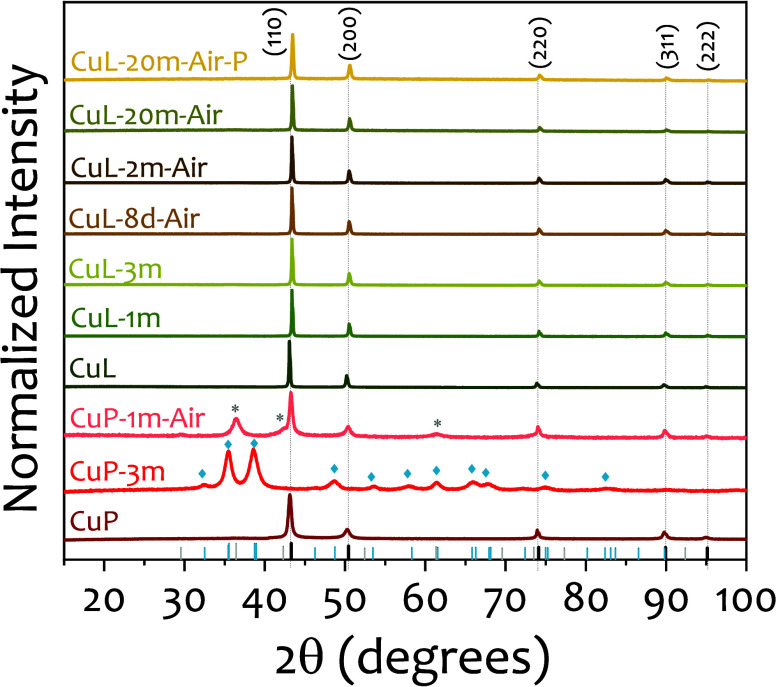
Evolution of the PXRD diffractograms of the samples under different time and storage conditions. PXRD patterns for Cu (PDF 00-004-0836), CuO (PDF 00-041-0254), and Cu_2_O (PDF 00-005-0667) are represented in in black, blue, and gray, respectively. Symbols * and ⧫ indicate the indexed Cu_2_O and CuO diffraction peaks, respectively. Sample names indicate the storage duration in days (d) or months (m) (e.g., 8d for 8 days, 1m for one month). Samples stored in ethanol carry no additional label; those labeled “Air” were stored as dry powders in ambient conditions; samples marked “P” refer to powders compacted into pellets and stored in air.

For the CuP samples, rapid oxidation was observed when stored in ethanol immediately after synthesis, with diffraction peaks corresponding solely to copper oxide (CuO) and no detectable metallic copper phase. When exposed to ambient air, the oxidation process was slower; metallic copper peaks remained detectable after one month, although oxidation was still evident. In contrast, CuL samples showed remarkable stability under both storage conditions (e.g., ethanol or exposed to ambient air). No signs of oxidation were observed by PXRD even after four months in ethanol or two months in ambient air. Notably, the PXRD diffractogram of a CuL sample stored for 20 months, both in powder and in pellet form, also showed no detectable oxidation, further confirming the effectiveness of the lignin coating as a long-term barrier regardless of the storage environment.

To complement the phase information provided by PXRD results with a surface-sensitive probe, XPS analysis (Cu 2p region) was performed on CuL pellets and compared with two commercial copper nanoparticle references: CCu (uncapped) and CCuE (coated with ethylene glycol following a previously reported reductive coating protocol^39^), shown in Figure 7. Importantly, these XPS measurements were carried out after approximately 24 months of the pellets being stored in ambient conditions. The spectra reveal that the CuL sample retains a predominantly metallic Cu surface contribution, whereas the relative metallic signal decreases for CCuE and is lowest for CCu, which exhibits the highest fraction of CuO at the surface. This trend is consistent with the role of lignin in limiting surface oxidation over extended storage times. Using the overlayer attenuation model described above (see Section 2.4, eq 1), the estimated CuO shell thicknesses after ∼ 24 months in air were ∼ 0.74 nm (CuL), ∼0.97 nm (CCuE) and ∼1.95 nm (CCu). Although XPS was measured only at this final time point (and therefore does not provide a full time-resolved kinetics), these values provide a quantitative metric for the markedly reduced extent of surface oxidation in the lignin-capped sample, consistent with the PXRD-based long-term stability trends. It should be noted that XPS probes only the near-surface region (typically ∼5 to 10 nm) and therefore can detect thin surface oxides and/or amorphous passivation layers that may not be visible by PXRD, which reflects long-range crystallinity and the bulk phase composition. Accordingly, crystalline secondary phases below the typical laboratory PXRD detection limit (approximately 2–5 wt %, depending on crystallinity and measurement conditions) may remain undetectable in the diffraction patterns.

**Figure 7:**
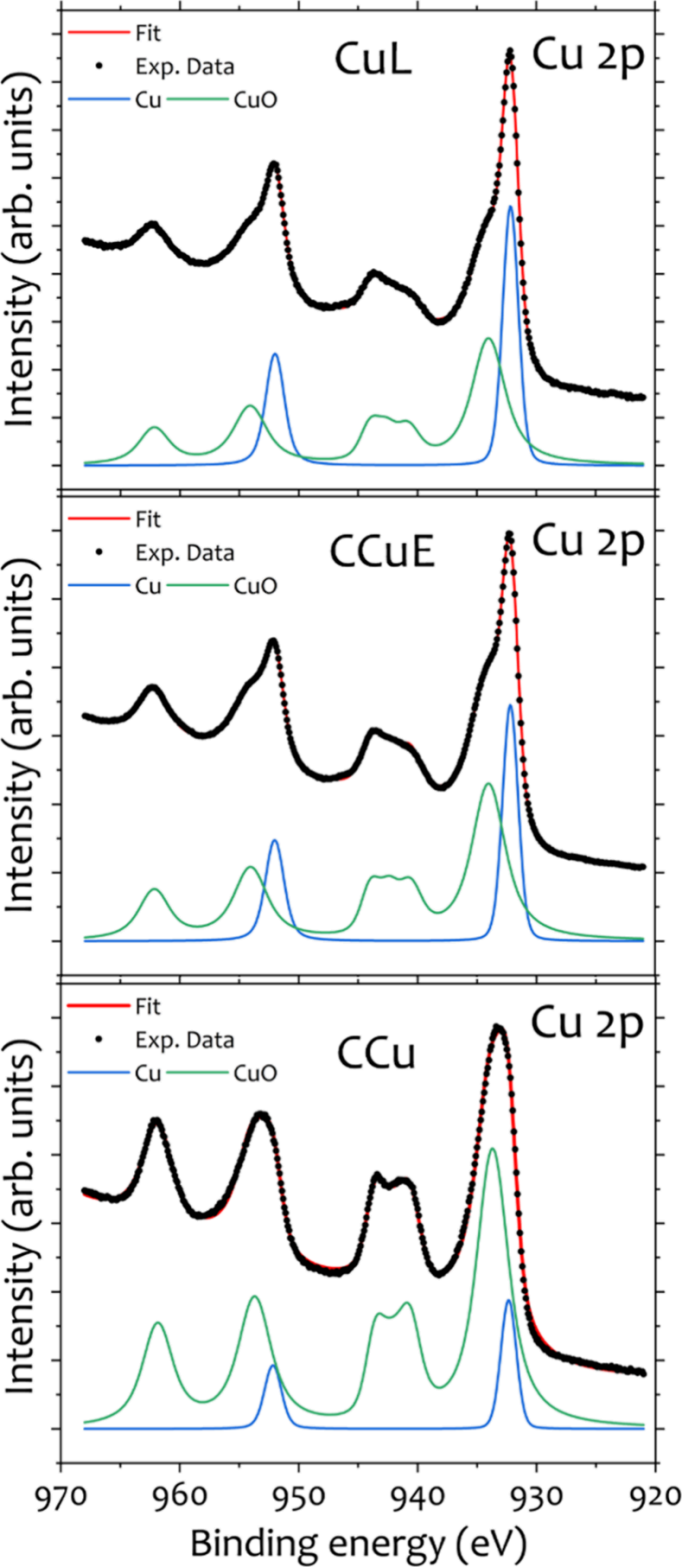
XPS Cu 2p core-level spectra of CuL (top), CCuE (middle), and CCu (bottom) samples. Experimental data (symbols), overall fit (solid line), and deconvoluted contributions from metallic Cu and CuO are shown. The spectra were fitted using a two-component model accounting for metallic copper and copper oxide contributions. A higher relative intensity of the metallic Cu component is observed for CuL, followed by CCuE, while CCu shows the largest contribution from CuO.

Overall, the combined PXRD and XPS results indicate that lignin capping preserves the metallic character of CuL during storage, both in dispersion and as dry material, supporting its suitability as a stable ink precursor. This robust and persistent protection is particularly valuable for ensuring the integrity of copper nanoparticle-based inks prior to formulation and deposition onto substrates. However, further studies will be required to evaluate the oxidation resistance and long-term performance of formulated inks under application-relevant conditions, such as elevated temperature and humidity. Still, the demonstrated shelf-stability over months (up to 20–24 months) significantly improves the storage potential of the material and provides a practical window for downstream processing, making it better suited for subsequent handling and use in printed electronics. This is specially promising for applications with limited functional lifetimes, such as single-use biosensors, wearable medical patches, or RFID tags, where midterm stability in powder or ink form is more critical than extended operation after printing.

### 3.3 Reproducibility of the Synthesis

To evaluate the reproducibility of the optimized copper nanoparticle synthesis, five independent batches were prepared under identical conditions using lignin as the capping agent. Reproducibility is a critical factor in nanoparticle synthesis, as slight variations in reaction conditions can significantly affect particle size, composition, and phase purity. Therefore, both TEM and PXRD analyses were performed on the five replicates (R1–R5) to assess consistency in morphology and crystal structure. TEM analysis (see Supplementary Figure S2) revealed comparable morphology and particle size distributions across all repetitions, with average diameters ranging from 102 to 181 nm. When considered collectively, the five repeats yield an overall average particle size of 132.8 ± 30.7 nm (*n* = 5), indicating a moderate batch-to-batch dispersion rather than a systematic drift. Although some variability in polydispersity was observed, the overall size range and dispersion remained consistent, supporting the robustness of the synthetic approach. Additionally, PXRD patterns (see Supplementary Figure S3) confirmed the exclusive formation of metallic copper in all replicates, with no detectable peaks corresponding to oxide phases. Table 2 summarizes the results obtained from TEM, PXRD, and Rietveld refinement for the CuL sample and five independent synthesis repetitions (R1–R5). The consistency across repetitions confirms the reproducibility of the synthesis protocol. Overall, these findings demonstrate that the microwave-assisted polyol method using lignin as a capping agent enables the reliable production of stable Cu NPs with comparable structural and morphological features. Although some batch-to-batch variation in average particle size was observed, this level of variability is typical for colloidal metal nanoparticle syntheses, particularly under rapid and scalable conditions. Even small deviations in weighed precursor/additive masses (within balance tolerances), minor differences in mixing history, or variations in the effective heating profile during the initial reduction stage can shift the nucleation-to-growth balance and thereby influence the final mean size. Further optimization of parameters such as mixing efficiency, precursor concentration, and lignin characteristics may help to improve size uniformity in future work.

**Table 2:** Summary of the Average Nanoparticle Diameter (*D*_TEM_), Crystallite Size (*D*_PXRD_), and the Cubic Cell Parameter *a* Obtained from Rietveld Refinement together with Their Respective Standard Deviations for the CuL Sample and Five Independent Synthesis Repetitions (R1–R5) with the Average Figures and Their Standard Deviation

sample	*D*_TEM_ (nm)	σ_TEM_ (nm)	*D*_PXRD_ (nm)	σ_PXRD_ (nm)	*a* value (Å)	σ_a_ (Å)
CuL	114	28	64	42	3.614	0.0001
R1	181	143	42.3	19.9	3.617	0.0003
R2	117	51	89.8	70.1	3.617	0.0001
R3	102	30	40.0	23.9	3.618	0.0002
R4	160	162	45.4	21.8	3.617	0.0004
R5	123	89	38.7	18.6	3.617	0.0002
average	132.8 ± 30.7		53.4 ± 20.1		3.617 4 ± 0.0001	

### 3.4 Conductivity Measurements

The electrical conductivity of the synthesized Cu NPs was evaluated by pressing dried powder samples into pellets several weeks after synthesis. This approach was selected as a rapid, formulation-independent benchmark to compare the intrinsic electrical performance of the different nanoparticle powders under identical consolidation and measurement conditions. In this configuration, compaction increases interparticle contact and may induce partial necking/sintering, thereby enhancing conductivity. We note that, while pellet conductivity provides a controlled comparison of consolidated nanoparticle networks, printed-electronics performance is ultimately assessed via sheet resistance of printed features, which depends on additional variables (ink formulation and dispersion stability, rheology, printing parameters, film thickness, substrate interactions, and post-treatment/sintering protocols).

Two samples from this work, CuP (PVP-capped) and CuL (lignin-capped), were compared against two commercial copper nanoparticle references: CCu (uncapped) and CCuE (coated with ethylene glycol following a protocol already published). These commercial materials were included as practical benchmarks of readily available Cu nanopowders, enabling a direct, application-relevant comparison under identical pellet-processing and measurement conditions. The conductivity values are summarized in Figure 8.

**Figure 8:**
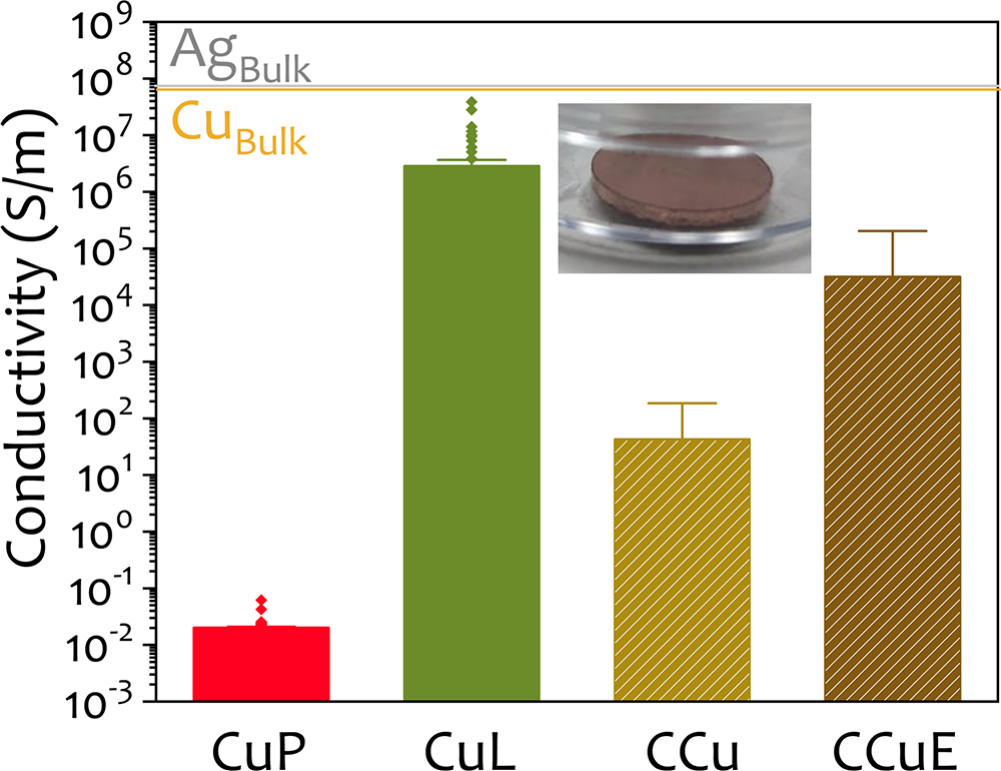
Conductivity measurements of the synthesized samples (CuP and CuL, with PVP40 and lignin as capping agents, respectively), and the commercial ones (CCu and CCuE, with no capping agent and ethylene glycol, respectively) pressed into pellets. The inset shows a representative image of one of those produced pellets.

Among all samples, CuP exhibited the lowest conductivity, with a mean value 2.00 × 10^–2^ S/m, likely due to partial oxidation observed previously, which hinders effective interparticle connectivity. The commercial sample CCu showed better performance, reaching 65.1 S/m, though still limited by significant oxidation, as confirmed by its PXRD pattern (Figure S4, Supporting Information), which displays intense peaks from copper oxide phases. Interestingly, the conductivity of CCu improved markedly upon treatment with ethylene glycol. The EG-coated version (CCuE) reached a conductivity of 5.88 × 10^4^ S/m, relating with a visibly reduced oxide fraction, as seen in its corresponding PXRD spectrum (Figure S4 in Supporting Information). This enhancement aligns with previous reports showing that protective or stabilizing agents like EG can mitigate oxidation in metallic nanopowders, thereby improving their electrical performance.^39^

The highest conductivity was achieved by the lignin-stabilized sample (CuL), with a mean value of 3.83 × 10^6^ S/m, corresponding to a resistivity of 26.1 μΩ·cm. This performance not only surpasses the other synthesized and commercial samples but also underscores the effectiveness of lignin in preserving the metallic copper phase and promoting efficient interparticle electrical contact. Although lignin is electrically insulating in bulk, its role here is primarily to passivate the Cu surface and inhibit oxidation during synthesis and storage rather than to act as an electronically active phase. In pressed pellets (3 tons, 5 min), electrical transport is dominated by interparticle junctions in the compacted Cu–Cu network; accordingly, the high conductivity of CuL suggests that any residual lignin is confined to thin interfacial regions that do not prevent effective particle–particle connectivity.

As discussed in Section 3.2, where the surface composition was evaluated by XPS, CuL pellets retain a predominantly metallic Cu contribution compared with CCuE and CCu. Because electrical transport in pressed pellets is governed by interparticle junctions, even very thin surface oxide layers can act as insulating barriers and sharply reduce the effective conductivity; accordingly, the XPS trend is consistent with the measured conductivity ranking (CuL > CCuE > CCu). For reference, the bulk conductivities of pure copper and silver are approximately 5.96 × 10^7^ and 6.30 × 10^7^ S/m, respectively.^53^ Although the conductivity of CuL remains approximately 1 order of magnitude lower than that of bulk silver, it is more than sufficient for a wide range of applications in printed and flexible electronics, particularly where cost is the primary concern. Moreover, in many emerging technologies, additional factors such as scalability, material abundance, and environmental impact take precedence over maximizing conductivity. Copper-based inks have already proven effective in areas such as RFID antennas, smart packaging, and disposable sensors, where slightly lower conductivities are acceptable without compromising device performance.^18,54,55^ Taken together, these advantages combined with the oxidation resistance and green synthesis route of CuL position it as a strong candidate for next-generation, eco-friendly conductive inks.

## 4 Conclusions

An economical and simple green chemical reduction method was developed for the synthesis of nanometric copper particles through a microwaved assisted polyol method and the use of H_2_PO_4_ as an environmentally friendly reductant and lignin as a capping agent. After a short homogenization phase, nanoparticle formation takes place during a final microwave irradiation step of only 5 min. This combination enabled the rapid formation of metallic Cu NPs with an average size of approximately 150 nm.

Notably, the use of lignin provided exceptional long-term stability to the Cu NPs. The metallic phase was preserved for up to four months in ethanol and 20 months in powder form stored in air, highlighting the lignin’s effectiveness in protecting the nanoparticles from oxidation. Surface-sensitive XPS (Cu 2p) measurements performed on CuL pellets after ∼24 months of storage further corroborated this stability, revealing a predominantly metallic Cu surface contribution and a lower CuO fraction compared with commercial Cu nanoparticle references (uncapped and EG-coated). This extended stability significantly enhances the material’s practicality for downstream applications. Importantly, the lignin-capped Cu NPs exhibited excellent electrical conductivity (3.83 × 10^6^ S/m), confirming their potential as high-performance conductive materials. This performance highlights lignin’s dual role as both a green stabilizer and a surface modifier that enhances interparticle connectivity while preserving the metallic character of the nanoparticles.

The overall process is aligned with green chemistry principles and supports the shift toward more sustainable manufacturing practices in electronics. Future work will include quantitative sustainability metrics, such as life-cycle assessment and comparative energy auditing, to further benchmark this approach. Meanwhile, the present study supports a greener positioning at the protocol level, based on renewable lignin stabilization, avoidance of highly hazardous reductants and inert atmospheres, and substantially shortened processing enabled by microwave heating. The simplicity, short processing time, and scalability of this synthesis approach make it well-suited for industrial-scale production of copper powders. These materials are particularly promising as functional fillers in conductive inks or pastes designed for flexible substrates, contributing to the advancement of next-generation printed and flexible electronics with a lower environmental footprint.

## Supplementary Material



## Notes

Table S1, representative recent reports on Cu nanoparticle stabilization strategies with synthesis route/heating approach, typical size, and oxidation stability over time; Table S2, synthesis parameters for Cu nanoparticle samples analyzed; Figure S1, SEM–EDX of CuL with P 2p XPS region showing no detectable phosphorus; Figure S2, TEM images and size statistics for CuL and reproducibility batches (R1–R5); Figure S3, PXRD patterns for lignin-capped Cu NPs from the reproducibility study with reference PDFs; and Figure S4, TEM and PXRD of commercial Cu nanoparticle references (CCu and CCuE) with indexed oxide peaks (PDF)
