# Supplementary material for: Lignin-Based Copper Nanoparticles for Green and Flexible Electronics
Source: ACS Appl Electron Mater. 2026 Mar 5;8(7):2901–12. doi: 10.1021/acsaelm.5c02668 (PMC13086123; doi:10.1021/acsaelm.5c02668)
Supplement: Supplementary file 1 [file el5c02668_si_001.pdf]

Supporting Information

Lignin-based copper nanoparticles for green and flexible electronics

María Salvador[1,2,*], Antonio Santana-Otero[1], Yilian Fernández-Afonso[1], Sabino Veintemillas-Verdaguer[1], André Van Zomeren[3], Salvador Bertran-Llorens[3], Alejandro Gutiérrez[4], María del Puerto Morales[1,*]

[1]*Instituto de Ciencia de Materiales de Madrid, ICMM/CSIC, C/Sor Juana Inés de la Cruz 3, 28049 Madrid, Spain*

[2]*Department of Physics, University of Oviedo, Campus de Viesques, 33204 Gijón, Spain.*

[3]*The Netherlands Organisation for Applied Scientific Research (TNO), Energy and Materials Transition, Biobased and Circular Technologies Group, P.O. Box 15, 1755 ZG Petten, The Netherlands*

[4]*Departamento de Física Aplicada and Instituto Nicolás Cabrera, Universidad Autónoma de Madrid, C/ Francisco Tomás y Valiente, 7, E-28049 Madrid, Spain*

*Corresponding author(s): María Salvador (m.salvador@csic.es), María del Puerto Morales (puerto@icmm.csic.es)

Table S1. Representative recent reports on copper nanoparticle stabilization strategies for conductive-material applications, grouped by stabilization family and summarized in terms of stabilizer chemistry, synthesis route/heating approach, typical particle size, and reported oxidation stability over time. Reported stability refers to the specific aging/storage protocol described in each reference (e.g., ambient storage or dispersion aging) and the corresponding analytical evidence (typically XRD).

Stabilization family	Ref. (Year)	Stabilizer	Synthesis route / heating	Typical size	Stability over time
**Biopolymer capping**	**This work**	**Lignin + NaH_2PO_2 reductant**	Microwave-assisted polyol	~114–150 nm	**Up to 4 months in ethanol; ~20–24 months as powder/pellet in air**
	Ref [1] (2025)	Lignin vs no lignin + NaOH	Continuous-flow microwave polyol	NR	**9 months:** without lignin oxide present from start; increases slightly but **<1.5%** by XRD; with lignin **no Cu_2O/CuO detected** (XRD)

**Small-molecule ligands (carboxylate/acid)**	Ref [2] (2022)	1-hexanoic acid (carboxylic-acid stabilization)	Paste/powder approach	~80 nm	NR
**Small-molecule ligands (N/O donors)**	Ref [3] (2025)	2-pyridinemethanol (2-HMP) thin organic interphase	Alcohol-phase reduction	20–30 nm	**~30 days storage stability** reported
**Small-molecule ligands (multifunctional acid)**	Ref [4] (2025)	Tartaric acid (dual reducing + capping), metal displacement	Mild displacement route (Al)	~3 nm	**~45 days storage** comparison (fresh vs aged XRD)
**Organic protective layer (heterocycles)**	Ref [5] (2023)	Organic protective agent (N-heterocycle type)	Liquid-phase reduction	~195 nm	**Air exposure series up to ~30 days** (XRD monitoring reported)
**Core–shell barrier**	Ref [6] (2023)	Cu@Ag core–shell	Oxidation mitigation via Ag shell	~100 nm	**≥60 days ambient exposure** with minimal oxidation reported
**Ionic/weak ligand capping + post-treatment aid**	Ref[7] (2021)	Citrate capping (+ sodium alginate sintering aid)	Aqueous wet-chemical reduction	~300 nm (SEM; not statistically quantified)	**8 weeks:** slight oxidation of the film
**Small-molecule/additive-controlled (high-reactivity reductant)**	Ref[8] (2025)	Small-molecule/additive-controlled stabilization. Uses **$NaBH_4$**;	Alcohol-phase reduction	~8.5 nm	**3 months (ambient):** dispersion aging + XRD phase check; **no Cu oxide peaks reported** after 3 months

Table S2. Summary of some of the synthesis parameters for the copper nanoparticle samples analyzed in this study.

Sample	Cu Salt $CuCl_2 \cdot 2H_2O$ (mg)	Reducing agent NaH_2PO_2 (mg)	Capping agent	(mg)	Solvent (EG) (mL)	Temperature (ºC) Initial	Reaction
Cu	1500.2	1653.5	NA		20	70º	160º
CuP	1500.3	1653.5	PVP40	177.8	20	70º	160º
CuL	1500.3	1653.5	Lignin	177.8	20	70º	160º
CuL^+	1500.3	1653.5	Lignin	304.0	20	70º	160º
CuL^-	1500.3	1653.5	Lignin	90.0	20	70º	160º
CuP^+	1500.3	1653.5	PVP40	350.0	20	70º	160º
CuP^-	1500.3	1653.5	PVP40	90.8	20	70º	160º
CuL – 5 min	1500.3	1653.5	Lignin	177.8	20	70º	160º
CuL – 45 min	1500.3	1653.5	Lignin	177.8	20	70º	160º
CuL – 20 min NH	1500.3	1653.5	Lignin	177.8	20	-	160º

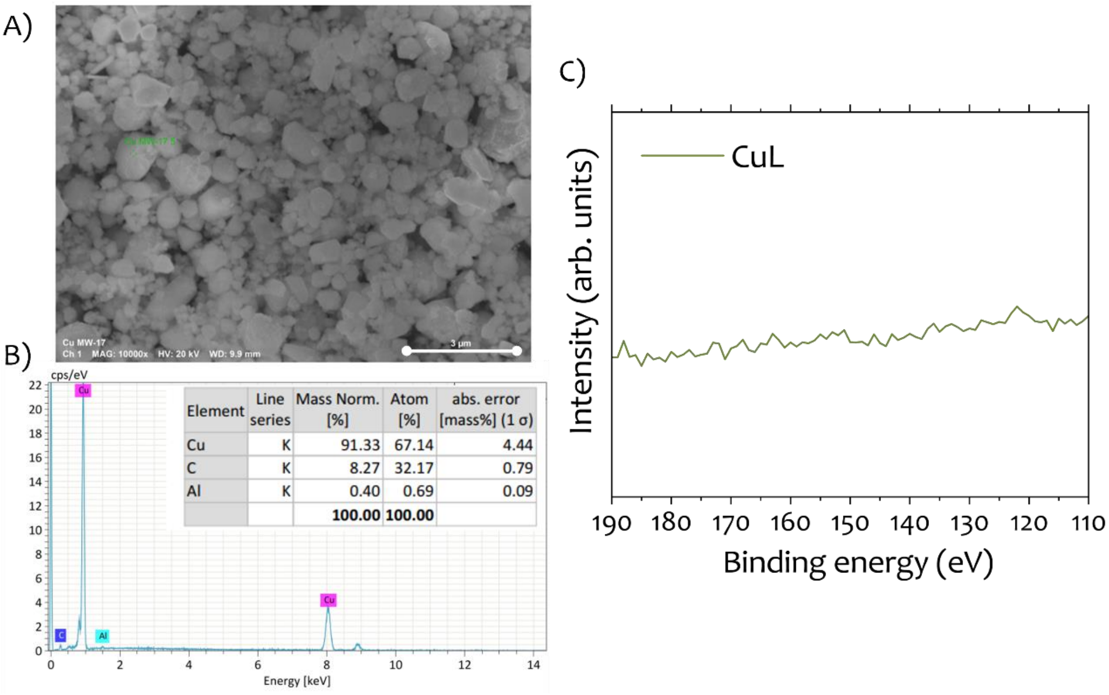

Element	Line series	Mass Norm. [%]	Atom [%]	abs. error [mass%] (1 σ)
Cu	K	91.33	67.14	4.44
C	K	8.27	32.17	0.79
Al	K	0.40	0.69	0.09
		**100.00**	**100.00**	

Figure S1. SEM–EDX analysis of the CuL sample. A) SEM image showing the morphology of the lignin-capped copper nanoparticles. B) EDX spectrum confirming the elemental composition of the sample. The inset table shows the quantified atomic percentages of the detected elements. C) XPS spectrum of the P 2p region for the CuL sample. No detectable phosphorus-related peaks are observed.

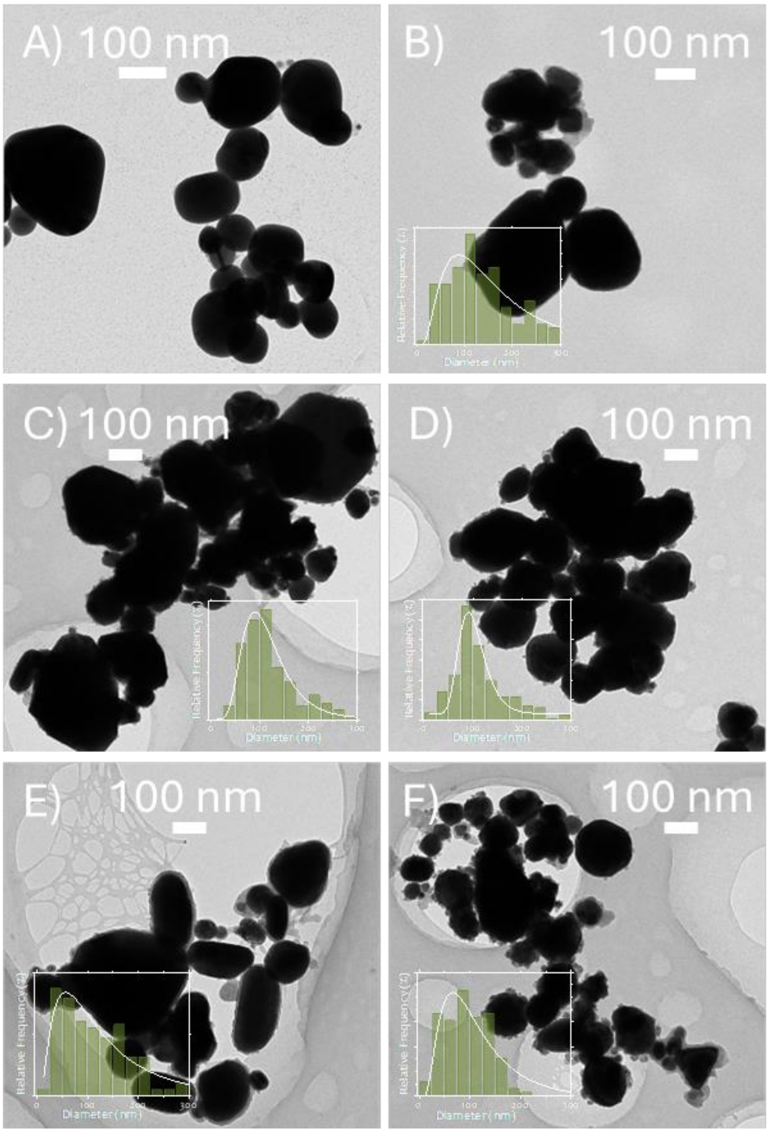

Figure S2. TEM images of the Cu NPs synthesized in the presence of lignin: A) CuL, and synthesis repetitions B) R1, C) R2, D) R3, E) R4, and F) R5. TEM images of the A) CuL nanoparticles and five independent synthesis repetitions under identical conditions: B) R1 (181 ± 143 nm), C) R2 (117 ± 51 nm), D) R3 (102 ± 30 nm), E) R4 (160 ± 162 nm), and F) R5 (123 ± 89 nm). The reported values in parentheses indicate the mean particle diameter and its standard deviation as obtained from TEM analysis.

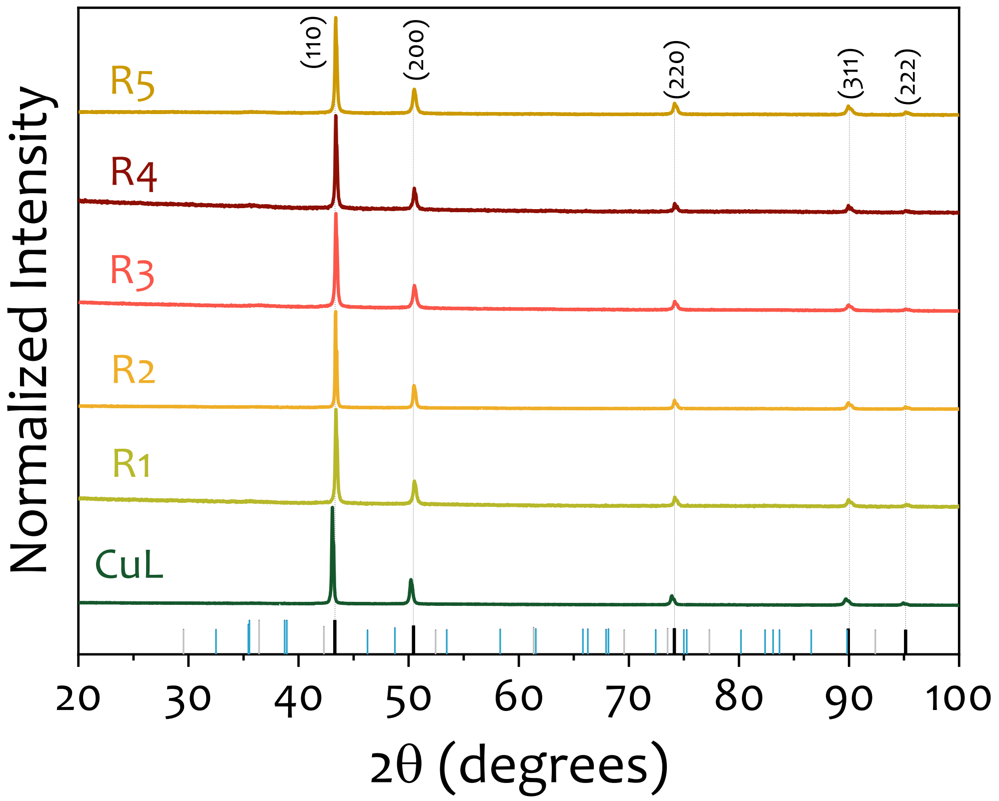

Figure S3. Results of powder X-ray diffraction analysis of the Cu NPs prepared in the presence of lignin and obtained in the reproducibility study. Reference patterns for Cu (PDF 00-004-0836), CuO (PDF 00-041-0254), and Cu_2O (PDF 00-005-0667), represented in black, blue, and gray, respectively.

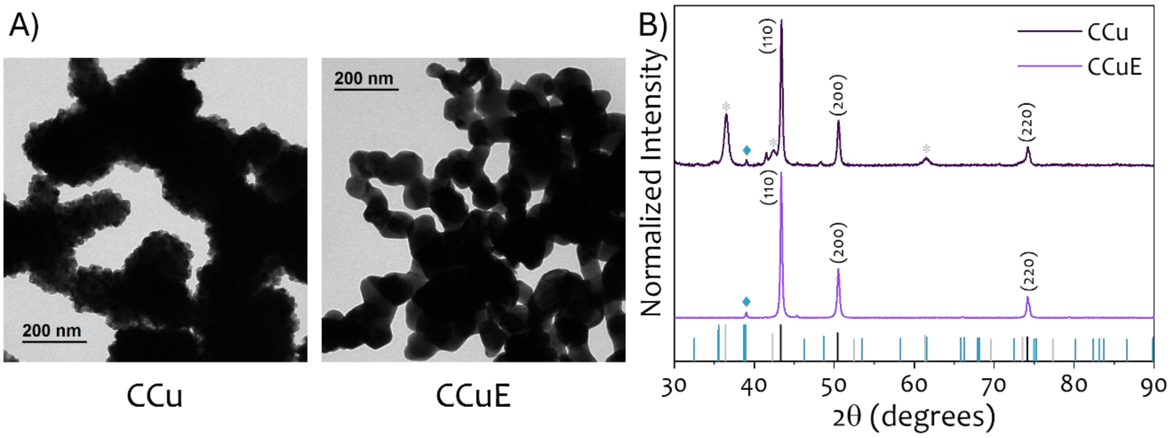

Figure S4. A) TEM images of the commercial copper nanoparticles CCu (uncapped) and CCuE (coated with ethylene glycol). B) Results of powder X-ray diffraction analysis of CCu and CCuE and comparison with XRD patterns for Cu (PDF 00-004-0836), CuO (PDF 00-041-0254), and Cu_2O (PDF 00-005-0667), represented in black, blue, and gray, respectively. Symbols * and ♦ indicate the indexed Cu_2O and CuO diffraction peaks, respectively.

References

(1) Santana-Otero, A.; Fernández-Afonso, Y.; Salvador, M.; Van Zomeren, A.; Bertran-Llorens, S.; Morales, M. del P.; Veintemillas-Verdaguer, S. Copper-Based Conductive Nanoinks: Scalable Synthesis via Continuous-Flow Microwave-Assisted Polyol Process. *ENERGY & ENVIRONMENTAL MATERIALS n/a* (n/a), e70164. https://doi.org/10.1002/eem2.70164.

(2) Tokura, R.; Tsukamoto, H.; Tokunaga, T.; Nguyen, M. T.; Yonezawa, T. The Role of Surface Oxides and Stabilising Carboxylic Acids of Copper Nanoparticles during Low-Temperature Sintering. *Mater. Adv.* **2022**, *3* (12), 4802–4812. https://doi.org/10.1039/D1MA01242H.

(3) Zhang, J.; Tian, F.; Wang, J.; Chen, H.; Li, M. A High-Performance Nano-Copper Paste with Good Oxidation Resistance. *Journal of Materials Research and Technology* **2025**, *38*, 1336–1341. https://doi.org/10.1016/j.jmrt.2025.08.010.

(4) Kandikonda, R.; Murugadoss, G.; Venkatesh, N.; Subbaraj, S. S. V.; Palani, D.; Thota, S.; Rajaboina, R. K.; Divi, H.; Dhayalan, M.; Phanumartwiwath, A.; Mallu, C. R.; Khanapuram, U. K. Redox-Driven Synthesis of Stable Copper Nanoparticles via Metal Displacement and Their Application in Organic Dye Degradation. *Mater. Adv.* **2025**, *6* (24), 9575–9589. https://doi.org/10.1039/D5MA00769K.

(5) Zhang, Y.; Yu, X.; Chen, Z.; Wu, S.; Lai, H.; Ta, S.; Lin, T.; Yang, G.; Cui, C. Synthesis of Imidazole-Compound-Coated Copper Nanoparticles with Promising Antioxidant and Sintering Properties. *Micromachines* **2023**, *14* (11), 2079. https://doi.org/10.3390/mi14112079.

(6) Li, G.; Yu, X.; Zhang, R.; Ouyang, Q.; Sun, R.; Cao, L.; Zhu, P. Facile Preparation of Monodisperse Cu@Ag Core–Shell Nanoparticles for Conductive Ink in Printing Electronics. *Micromachines* **2023**, *14* (7), 1318. https://doi.org/10.3390/mi14071318.

(7) Sarwar, N.; Choi, S. H.; Dastgeer, G.; Humayoun, U. B.; Kumar, M.; Nawaz, A.; Jeong, D. I.; Zaidi, S. F. A.; Yoon, D. H. Synthesis of Citrate-Capped Copper Nanoparticles: A Low Temperature Sintering Approach for the Fabrication of Oxidation Stable Flexible Conductive Film. *Applied Surface Science* **2021**, *542*, 148609. https://doi.org/10.1016/j.apsusc.2020.148609.

(8) Zhang, P.; Sun, Q.; Fang, S.; Guo, H.; Liu, K.; Zhang, L.; Zhu, Q.; Wang, M. Fabrication of Nano Copper Highly Conductive and Flexible Printed Electronics by Direct Ink Writing. *ACS Appl. Mater. Interfaces* **2025**, *17* (1), 1847–1860. https://doi.org/10.1021/acsami.4c14225.
